# Multiple component interventions for preventing falls and fall-related injuries among older people: systematic review and meta-analysis

**DOI:** 10.1186/1471-2318-14-15

**Published:** 2014-02-05

**Authors:** Victoria A Goodwin, Rebecca A Abbott, Rebecca Whear, Alison Bethel, Obioha C Ukoumunne, Jo Thompson-Coon, Ken Stein

**Affiliations:** 1 PenCLAHRC, University of Exeter Medical School, Veysey Building, Salmon Pool Lane, Exeter EX2 4SF, UK

**Keywords:** Falls, Systematic review, Aged

## Abstract

**Background:**

Limited attention has been paid in the literature to multiple component fall prevention interventions that comprise two or more fixed combinations of fall prevention interventions that are not individually tailored following a risk assessment. The study objective was to determine the effect of multiple component interventions on fall rates, number of fallers and fall-related injuries among older people and to establish effect sizes of particular intervention combinations.

**Methods:**

Medline, EMBASE, CINAHL, PsychInfo, Cochrane, AMED, UK Clinical Research Network Study Portfolio, Current Controlled Trials register and Australian and New Zealand Clinical Trials register were systematically searched to August 2013 for randomised controlled trials targeting those aged 60 years and older with any medical condition or in any setting that compared multiple component interventions with no intervention, placebo or usual clinical care on the outcomes reported falls, number that fall or fall-related injuries. Included studies were appraised using the Cochrane risk of bias tool. Estimates of fall rate ratio and risk ratio were pooled across studies using random effects meta-analysis. Data synthesis took place in 2013.

**Results:**

Eighteen papers reporting 17 trials were included (5034 participants). There was a reduction in the number of people that fell (pooled risk ratio = 0.85, 95% confidence interval (95% CI) 0.80 to 0.91) and the fall rate (pooled rate ratio = 0.80, 95% CI 0.72 to 0.89) in favour of multiple component interventions when compared with controls. There was a small amount of statistical heterogeneity (I^2^ = 20%) across studies for fall rate and no heterogeneity across studies examining number of people that fell.

**Conclusions:**

This systematic review and meta-analysis of randomised controlled trials found evidence that multiple component interventions that are not tailored to individually assessed risk factors are effective at reducing both the number of people that fall and the fall rate. This approach should be considered as a service delivery option.

## Background

Falls are a common problem affecting older people, with a third of those aged 65 and over, and half of those aged over 85, falling each year [1]. The consequences of falls are disability, reduced quality of life and financial costs to individuals and society [2]. The UK National Health Service (NHS) is reported to spend around £1.7 billion each year on falls [3]. As a consequence there has been a wealth of research undertaken to establish how best to prevent falls with interventions including exercise, home safety modifications and education [4]. These interventions have been categorised into one of the following three combinations: [5]

a) Single interventions e.g. exercise;

b) Multifactorial interventions where two or more individually tailored interventions are provided following a risk assessment e.g. one person may receive exercise and home hazard modification whereas another may receive home hazard and medication modifications; and,

c) Multiple component interventions, where participants receive a fixed combination of two or more interventions e.g. exercise and Vitamin D.

There is increasing evidence from meta-analyses for the effectiveness of single interventions, such as exercise, at reducing the rate of falls in community-dwelling [4] and mixed populations [6]. Home modifications have also been found to be effective at reducing fall risk [7]. Whilst combining interventions that are effective on their own might therefore seem intuitive, the evidence for combined interventions (multifactorial and multiple component as described above) is less clear. Multifactorial interventions, which require an individually tailored approach, have been shown in a meta-analysis to reduce the rate of falls [4] but there remains uncertainty in relation to reducing the number of those that fall [8]. Indeed, this is supported by a recently updated Cochrane review, including more than 13,000 participants which observed no benefit in a reduction in the number that fell [4]. These two reviews and meta-analyses [4,8] reported high levels of heterogeneity (I-squared between 60% and 69%) in the meta-analyses relating to number of people that fall, although this variation was not explained by baseline fall risk and intensity of interventions. That said, multifactorial interventions are the recommended approach for falls prevention in the UK [9] whereas multiple component interventions on the other hand, which do not necessitate an individual assessment, and might therefore be an alternative approach, have not been extensively evaluated. Whilst Gillespie and colleagues included multiple component interventions as part of their review, their synthesis was narrative with each study reported separately due to the variety of combined interventions undertaken [4]. Of the included studies that were effective, all but one included exercise but the omission of any summary data across the studies as a whole leaves an unclear picture as to the effectiveness of multiple component fall prevention programmes.

The aim of this review was to establish the effectiveness of multiple component interventions, as defined by Lamb et al. [5] targeting older people, on (a) number of people that fell, (b) fall rates, and (c) number that sustained a fall-related injury, including an exploration of between-trial variability.

## Methods

The review was conducted following the general principles of the NHS Centre for Reviews and Dissemination [10]. A pre-defined protocol was developed following consultation with topic and method experts and is available from the Peninsula Collaboration for Applied Research and Care (PenCLAHRC) website (http://clahrc-peninsula.nihr.ac.uk/multi-component-interventions-for-preventing-falls-and-fall-related-injuries-among-older-people--sys.php).

### Literature search and eligibility criteria

The search strategy from Gillespie et al. [11] was updated to run from May 2008 to August 2013 (Table 1). The search strategy was applied in the following databases: Medline In-Process, EMBASE, CINAHL, PsychInfo, Cochrane (CDSR, DARE, CMR, HTA and EED) and AMED. Clinical trial databases were also searched including Cochrane Central, UK Clinical Research Network Study Portfolio, Current Controlled Trials Register and the Australian and New Zealand Clinical Trials Register. Studies referenced by the Cochrane reviews of fall prevention targeting whole populations, [12] community-dwelling older people [4] and those in hospitals and care homes [13] were also considered as were the reference lists of included studies. No language restrictions were imposed.

**Table 1:** Search strategy (Ovid MEDLINE(R) 1946 to July week 4 2013)

	
1	Accidental falls/(12522)
2	(Falls or faller$1 or fallen).tw. (27753)
3	1 or 2 (33935)
4	Exp Aged/ (2056836)
5	(Senior$1 or elderly or older).tw. (335684)
6	4 or 5 (2182495)
7	Randomized controlled trial.pt. (319542)
8	Controlled clinical trial.pt. (83511)
9	Randomized.ab. (224781)
10	Placebo.ab. (128471)
11	Randomly.ab. (162506)
12	Trial.ab. (231806)
13	Groups.ab. (1073150)
14	3 and 6 (13056)
15	7 or 8 or 9 or 10 or 11 or 12 or 13 (1570937)
16	Humans.sh. (12063808)
17	15 and 16 (1190697)
18	14 and 17 (2966)
19	Limit 18 to ed=20080511-20120223 (968)

Randomised controlled trials (RCTs) were included if they compared multiple component interventions for fall prevention on fall rate, number of fallers or fall-related injuries in people aged 60 years or over (or those described as elderly, seniors or older people) with any medical condition, with no intervention, placebo or usual care. The latter is defined as the care patients would receive independent of the research. Eligible multiple component interventions had to include a fixed combination of two or more interventions including exercise, medication, surgery, management of urinary incontinence, fluid or nutritional therapy, psychological, environment or assistive technology, social environment or knowledge [5]. Studies that included younger participants, for example recruited on the basis of a medical condition such as a stroke or Parkinson’s disease, were included if the mean age minus one standard deviation was more than 60 years. Studies were excluded if they did not report fall-related outcomes or were reported only as abstracts.

### Study selection

Two reviewers independently screened all titles, abstracts and full texts and applied inclusion and exclusion criteria. Discrepancies were resolved by discussion with arbitration by a third reviewer where necessary.

### Data extraction

A standardised, piloted data extraction form based on that developed by Lamb and colleagues [5] was used to extract data (http://www.profane.eu.org/taxonomy.html). Data were extracted on population characteristics and fall risk status, intervention and comparator characteristics (setting, delivery, and description), fall-related outcome data (length of follow up, method of collection) and effect sizes. Those with prior falls and known fall risk factors (living in residential care, aged ≥75 years, or with impaired strength or balance were identified as high risk of falling) [14]. Data were extracted and quality assessed by one reviewer and checked by a second reviewer with discrepancies resolved by discussion and arbitration with another reviewer if necessary.

### Quality assessment

The methodological quality of each paper was assessed using the Cochrane risk of bias tool [15] by one reviewer, with judgements checked by a second. Detection bias was established separately for the assessment of falls and for the assessment of injuries/fractures. We assessed recall bias by examining the time period over which fall recall occurred. It has been reported that fall recall over a three month period is inaccurate [16] and that falls should be reported no less frequently than monthly [4].

### Data synthesis

Random effects meta-analysis was used to pool estimates of the effects of multiple component interventions using the DerSimonian-Laird method [17]. The effect on the falls rate was quantified using rate ratios and the effect on the number of people that fell was quantified using the risk ratio. Findings relating to the number of people sustaining fall-related injuries were reported descriptively. Meta-analysis was performed using the intervention effect estimates and standard errors using Review Manager (RevMan) Version 5.2 (http://ims.cochrane.org/revman). Heterogeneity across estimates was quantified using the I-squared statistic [18]. The p-value from the Q test was used to quantify evidence against homogeneity [18]. The likelihood of publication bias was examined using Funnel plots and Egger’s regression test [19] for asymmetry (using the *metabias* command in *Stata* software). Those studies that provided insufficient data to include in the meta-analysis were reported descriptively.

Where possible, numerator and denominator data were used to calculate study-specific estimates and standard errors. The numerator and denominator data for one paper [20] were extracted from a project report [21]. For five studies [22-26] the estimate of effect (risk, rate, or both) was taken directly from the paper and the standard error was calculated from the 95% confidence interval. For those studies that did not calculate and report fall risk and where sufficient raw data were reported, estimates of effect and 95% confidence intervals were calculated from these [24,26-30]. If outcome data were reported at multiple time points, the final endpoint was used in our analyses. Study-specific rate ratios were estimated using Poisson regression based on the number of events and the number of person-years in each trial arm as reported in the included papers. For some studies there was over-dispersion but because participant-level data were not available it was not possible to fit negative-binomial models.

Three studies [20,31,32] had two or more intervention arms that each comprised multiple component interventions and were separately compared with the control arm. To take account of the fact that some study-specific estimates used the same control arm we divided the information across the number of comparisons from the study [15]. When pooling rate ratios, the number of events and the number of person-years in the control arm were divided equally across the comparisons and when pooling risk ratios the number of fallers and the total sample size in the control arm were divided equally across the comparisons before calculating the standard errors that were used in the meta-analysis. Sensitivity analyses in which we used an alternative approach of inflating the variance of the study specific estimates up to four-fold to take account of multiple estimates from the same study provided almost identical results to the main analyses.

Three studies [20,28,33] used a cluster randomised trial design but two [20,28] did not take account of the correlation between participants within the same cluster. Sensitivity analyses were conducted in which the estimates related to these studies were removed. In addition, four sensitivity analyses were undertaken in which the variances of those estimates were inflated by design effects of 1.2, 1.5, 2 and 5 to allow for clustering. The pooled estimates and confidence intervals for the risk and rate ratios were essentially the same as those from the reported analyses.

## Results

**Figure 1:**
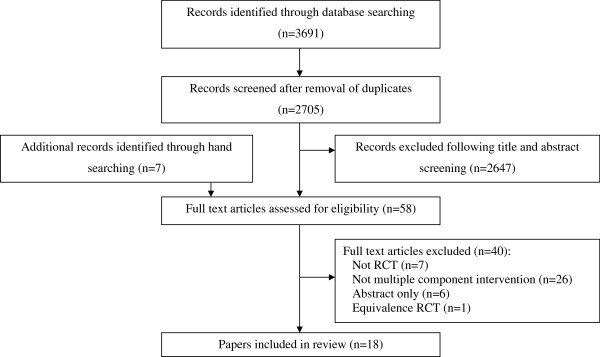
Flow chart of included studies.

The database search identified 3691 citations. Eighteen papers, representing seventeen studies were included (Figure 1). One paper [34] reported additional analyses from the study reported by Day et al. [32]. Two studies were not included in the meta-analysis due to insufficient data. One study [35] had too small a sample size (10 participants in each arm) to calculate valid confidence intervals for the risk ratio. Another study [36] combined results from single and multiple component interventions and it was not possible to extract the data for the multiple component intervention in relation to falls or fractures. Three studies [20,31,32] had several intervention groups, and thus, we had 19 estimates of effect to synthesise in the meta-analysis for fall risk ratio and 17 estimates in the meta-analysis of fall rate ratio. Four studies [25,31,36,37] reported injury data with two studies reporting fractures [25,36] and one reporting serious injuries (that comprised fractures and hospital admissions due to injury) [31]. One study provided no definition of fall-related injuries [37]. Egger’s test indicated little evidence of publication bias for the analyses of fall risk (p = 0.13) or fall rate (p = 0.76).

### Description of studies

A detailed description of the included studies can be found in Additional file 1. The trial designs included parallel group, [22-27,29,30,35,37,38] cluster [20,28,33] and factorial [31,32,36] design RCTs. The studies were undertaken in Australia, [20,22,25,32,33] the Netherlands, [24,26,38] New Zealand, [31] Switzerland, [35,36] Germany, [37] Sweden, [29] Taiwan [27,28] and the USA [23,30]. A total of 5034 participants were included, with sample sizes ranging from 24 [35] to 1,107 [32]. The mean age of participants in the included studies ranged from 69 to 86.4 years and the proportion of females ranged from 38% to 100%. Twelve studies [7,24-26,29-31,33,35-38] recruited only participants known to be at high risk of falling. Six studies targeted people with specific characteristics including visual impairment, [31] osteoporosis/osteopenia, [35] acute hip fracture, [36] stroke, [29] malnourishment [38] and foot problems [25].

Of the 14 studies that had only one intervention group, twelve included exercise as part of the multiple component intervention with additional interventions including medication (n = 3), continence management (n = 1), fluid or nutritional supplements (n = 2), psychological interventions (n = 4), environment or assistive technology (n = 5), and, knowledge (including written information, videos, lectures) (n = 10). Interventions categorised as ‘other’ included advice of medical risk factors (n = 1), vision improvement (n = 1) and sending an assessment summary along with falls guidelines to the general practitioner (n = 1). Of the two remaining studies that didn’t include exercise, one consisted of medication and nutritional supplements [38] and the other comprised medication and sunlight exposure [33]. Three studies [20,31,32] reported two or more different intervention groups of which one intervention group from a factorial study [32] did not include an exercise component but included home hazard interventions and vision improvement. Most interventions were delivered in community settings (n = 10). Two were delivered in outpatient clinics, one in a hospital, two in a care facility and three studies did not report where delivery took place. Controls were described as usual care (n = 9), information (n = 2), social visits (n = 2), and no intervention (n = 1) with three studies providing participants in both the controls and intervention arms with a standardised intervention.

Twelve studies reported falls as the primary outcome, with one study indicating both falls and fear of falling as primary outcomes [27]. Two studies indicated balance [29,35] and one reported activities of daily living [38] as the primary outcome with falls as a secondary outcome. One study [26] reported fear of falling as the primary outcome with falls reported as adverse events. Follow-up varied from three [38] to twenty four months [37].

### Quality assessment

The reporting of data relating to risk of bias was often lacking resulting in difficulty making clear judgements about potential risk (Additional file 2). Most studies had a low risk of recall bias having collected fall data over time periods of less than three months, whereas only seven studies reported allocation concealment and the remainder were at potential risk of selection bias.

### Outcomes

**Figure 2:**
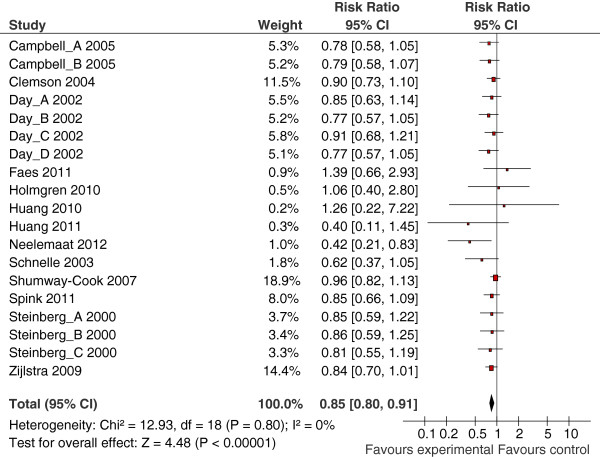
**Forest plot from the meta-analysis of multiple component interventions on number of fallers showing estimates of risk ratio, 95% confidence intervals and relative weight of each study.** Meta-analysis of intervention effect on number of fallers.

**Figure 3:**
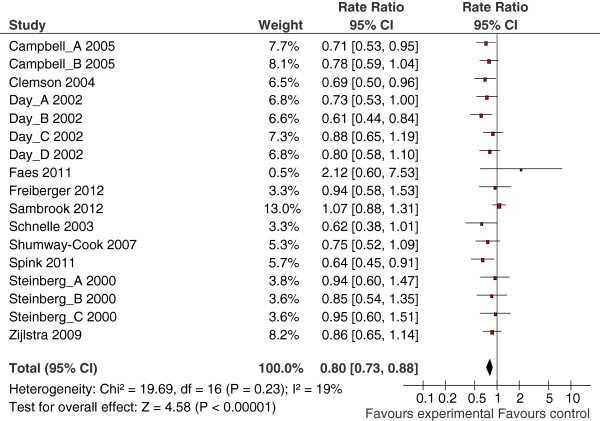
**Forest plot from meta-analysis of multiple component interventions on fall rates showing estimates of rate ratio, 95% confidence intervals and relative weight of each study.** Meta-analysis of intervention effects on fall rate.

A beneficial effect of multiple component interventions was observed for the number of people that fall (risk ratio =0.85, 95% CI 0.80 to 0.91, Figure 2) and fall rate (rate ratio =0.80, 95% CI 0.73 to 0.88, Figure 3). Two studies were undertaken in a care home setting, which may represent a very different population to those seen in a community or clinic setting. We therefore undertook a sensitivity analysis by removing the results of these papers from the meta-analyses but this made little difference (risk ratio = 0.86, 95% CI 0.80 to 0.92; rate ratio = 0.78, 95% CI 0.71 to 0.85). No heterogeneity was found across studies evaluating the number of people that fell (I2 =0%). There was a small amount of heterogeneity (I2 = 20%) across studies of fall rate. This heterogeneity was entirely due to one study [33] and sensitivity analysis made little difference (rate ratio = 0.77, 95% CI 0.70 to 0.85) and, therefore, it was not considered appropriate for sub-group analyses or exploration of variability between studies to be undertaken. Only one study [25] reported the number of people sustaining a fracture (risk ratio 0.14, 95% CI 0.02 to 1.15). Campbell and colleagues [31] reported serious injuries that included fractures, hospitalisation and injuries requiring stitches but there was no evidence at the 5% level of significance of an effect for either exercise and Vitamin D (risk ratio =0.98, 95% CI 0.25 to 3.84) or exercise, Vitamin D and home safety (risk ratio =2.7, 95% CI 0.89 to 8.17) versus control. One study [37] reported injurious falls but did not define this (incidence rate ratio = 1.02, 95% CI 0.54 to 1.95).

## Discussion

We assessed RCTs that compared multiple component interventions, where participants received a fixed combination of two or more interventions that were not individually tailored based on a fall risk assessment, with a control. We found that, overall, multiple component interventions were effective at reducing the number of people that fall and the fall rate but it remains unclear as to the benefits of preventing fall-related injuries. Undertaking in-depth assessments with individuals in order to establish individual risk is recommended in the UK as best practice [9] whereas a recent report by the US Preventative Services Task Force recommended that multifactorial assessment and individually tailored interventions are not undertaken as they provide only a small net benefit [39]. In addition, there has also been recent debate in the Journal of the American Geriatrics Society on the relative benefits of single versus multifactorial interventions to prevent falls among community dwelling older people [40,41]. The results of this review provide evidence for a third approach, having multiple components that target the population of interest rather than targeting individual risk factors, which may be potentially less resource intensive than multifactorial programmes as staff would not need to undertake in-depth, multifactorial assessment to establish individual risk factors.

### Comparison with existing literature

Although a number of systematic reviews have synthesised findings from single and individually tailored multifactorial fall prevention interventions, there has been limited evaluation of multiple component interventions as defined by Lamb and colleagues [5]. Gillespie et al. [4] reported the findings from 19 RCTs examining multiple component interventions, although studies were not pooled due to clinical heterogeneity of the interventions. They concluded that few multiple component interventions were effective. Ten of their studies were included in our review with the remainder being excluded as they did not meet pre-specified selection criteria on the basis of the study design (n = 2), being unpublished (n = 1), being published only as an abstract (n = 2), the intervention not being multiple component (n = 2) and controls not being inert in relation to preventing falls (n = 1). The findings of our review are supported by a Cochrane review of population-based interventions to prevent falls among older people [12] that comprised six non-randomised controlled studies. They concluded that interventions aimed at the population rather than individuals reduced fall-related injuries, with relative reductions ranging from 6% to 33%, suggesting that interventions that are not tailored to the individual can be effective. We found only two studies [30,33] undertaken with care home residents and therefore the review findings should be interpreted with some caution in relation to this population.

In our review, most of the comparisons included in the meta-analyses included exercise, indicating that exercise may be an important element of multiple component interventions, although we cannot conclude whether exercise is essential as some effective studies did not include exercise. This concurs with the findings from other systematic reviews [4,42]. Sherrington et al. [6] undertook a synthesis of 54 RCTs evaluating only the effect of exercise interventions on fall rate (rate ratio =0.84, 95% CI 0.77 to 0.91) and reported moderate heterogeneity (I^2^ = 56%) of which 64% was explained by studies that included balance training, a dose of > 50 hours duration and no walking programme. The actual impact of multiple interventions reported in our review, however, remains unclear. The added benefit we observed from additional interventions may not be dependent upon the type of intervention, just as long as it is appropriate to the target population. Whereas the findings from reviews evaluating multifactorial interventions, [4,8] where two or more individually tailored interventions are provided following a multifactorial risk assessment, indicated uncertainty of benefit in reducing the number of people that fall, the review reported here demonstrated benefit without individual tailoring. This supports fall prevention advice from the US that recommends exercise and Vitamin D supplementation [39] and findings from a review of population-based studies [12].

### Strengths and limitations

This review was undertaken following recommended methods with systematic and comprehensive searching without restriction based on language. However, the exclusion of studies reported only as an abstract may be considered a limitation. This was an *a priori* decision made at the protocol stage, to reduce publications that may not have been peer-reviewed.

The studies included in this review did not always report falls outcomes as recommended [43] and as such for some studies it was necessary to calculate effect sizes where data allowed. This, however, relies on secondary analysis based on reported data, as opposed to individual patient data, which may be subject to rounding error. That we had no access to participant-level data also meant that it was not possible to allow for over-dispersion when estimating study-specific rate ratios. Not all studies presented data in a way that could be used in the meta-analysis and were therefore only described. Injuries were rarely reported in the included studies, and could be considered a flaw in the original studies, and even when reported the definitions used were inconsistent.

The lack of observed statistical heterogeneity across studies examining the number of people that fell meant that we could not explore the potential sources of clinical heterogeneity using meta-regression techniques to identify the most effective combination of interventions. The heterogeneity across studies evaluating fall rate was due to a single study [33].

### Implications for research and practice

There are a number of potential treatment options to reduce falls amongst older people and those at risk of falling, such as single, multifactorial and multiple component interventions but it remains unclear as to whether one approach is superior to others as there have been no head-to-head comparisons.

International and UK guidelines [9,39,44] give conflicting recommendations as to whether older people that have fallen or are at high risk of falling should receive multifactorial assessment and individually tailored interventions. Our review, however, shows that multiple component interventions (that are not individually tailored) appear to be effective at reducing both the number of older people that fall and the number of falls across general older and high risk populations and should therefore be considered as an option for future service delivery, particularly as individually tailored programmes are resource intensive. Future research to compare these different approaches, particularly in terms of cost-effectiveness should be considered.

## Conclusions

This systematic review and meta-analysis of randomised controlled trials found evidence that multiple component interventions that are not tailored to individually assessed risk factors are effective at reducing both the number of people that fall and the fall rate. This approach should be considered as a service delivery option.

## Competing interests

The authors declare that they have no competing interests.

## Authors’ contributions

VG conceived the idea and developed the protocol and undertook screening and data extraction, analysis and prepared the manuscript. RW contributed to the protocol development and undertook screening and data extraction, and contributed to the manuscript. RA undertook screening, data extraction, and contributed to the manuscript. AB contributed to the protocol development and developed the search strategy, undertook screening, data extraction and contributed to the manuscript. JTC contributed to the protocol development and undertook screening and contributed to the manuscript. OU contributed to the protocol development and undertook analyses and contributed to the manuscript. KS contributed to the protocol development and contributed to the manuscript. All authors read and approved the final manuscript.

## Pre-publication history

The pre-publication history for this paper can be accessed here:

http://www.biomedcentral.com/1471-2318/14/15/prepub

## Supplementary Material

Additional file 1 Characteristics of Included Studies. Click here for file

Additional file 2 Risk of Bias of Included Studies. Click here for file
