# Supplementary material for: Multiple component interventions for preventing falls and fall-related injuries among older people: systematic review and meta-analysis
Source: BMC Geriatr. 2014 Feb 5;14:15. doi: 10.1186/1471-2318-14-15 (PMC3928080; doi:10.1186/1471-2318-14-15)
Supplement: Additional file 1 — Characteristics of Included Studies. [file 1471-2318-14-15-S1.docx]

Characteristics of Included Studies

|  | **Inclusion criteria** | **High fall risk population** | **Population characteristics** | **Total number of participants randomised** | **Intervention** | **Control** |
| --- | --- | --- | --- | --- | --- | --- |
| Steinberg  2000 | ≥50 | No | Mean age 69 (range 51-87); 79% female | 252 | Group A - education, monthly exercise group (15 months), home exercise video;  Group B - (A) +Home assessment/advice;  Group C - (B) + medical assessment/advice | Home safety presentation and information on risk factors |
| Day  2002 | ≥70; community dwelling | No | Mean age 76.1 (SD 5.0); 60% female | 1090 | Group A – weekly exercise class (15 weeks) and home hazard modification;  Group B - weekly exercise class (15 weeks)and vision correction;  Group C – home hazard modification and vision correction | Usual care |
| Schnelle  2003 | Nursing home resident; incontinence | Yes | 85% female | 190 | Daily exercise and mobility; regular toileting; regular fluids (8 months) | Usual care |
| Clemson  2004 | ≥70; fallen or felt at risk of falling | Yes | Mean age 78 (SD 5); 74% female | 310 | Weekly exercise and education class (7 weeks); occupational therapy (OT) home visit | Student OT home visit |
| Campbell  2005 | ≥75; severe visual impairment | Yes | Mean age 83.6 (SD 4.8), range 75-96; 68% female | 391 | Group A – home exercises (3 times a week for a year) supported by 5 visits by a physiotherapists + 2.5mg calciferol monthly;  Group B – (A) + home safety assessment/advice | Social visits by a researcher |
| Shumway-Cook 2007 | ≥65; community dwelling | No | Mean age 75.6 (SD 6.3), range 65-96; 77% female | 453 | Exercise group (3 times weekly for a year), education, falls risk assessment sent to general practitioner | Usual care |
| Swanenburg 2007 | ≥65; Osteoporosis or ostepaenia | Yes | Mean age 71.2 (SD 6.8); 100% female | 24 | Exercise group (3 times a week for 12 weeks) + 400-800IU Vitamin D3 and 500-1000mg calcium per day + daily protein drink | 400-800IU Vitamin D3 and 500-1000mg + home exercise leaflet |
| Zijlstra  2009 | ≥70; fear of falling; community dwelling | Yes | Mean age 77.9 (SD 4.8); 74% female | 540 | Weekly group cognitive behavioural therapy sessions for 8 weeks + group exercise (15 minutes a week for 6 weeks) + home exercises | Usual care |
| Bischoff-Ferrari 2010 | ≥65; acute hip fracture | Yes | Mean age 84 (range 65-99); 79% female | 173 | Vitamin D3 (2000 IU/day) plus 500mg/day calcium + 30 minutes daily inpatient physiotherapy + instruction on home exercises + daily home exercises following hospital discharge | Vitamin D3 (2000 IU/day) plus 500mg/day calcium + 30 minutes daily inpatient physiotherapy |
| Holmgren  2010 | ≥55; stroke in past 3-6 months | Yes | Mean age 78.5 (SD 7.5; 38% female | 34 | Exercise programme (6 times a week for 5 weeks) + home exercises (3 times a week for 3 months) + weekly fall education programme + home assessment visit | Education not related to falls |
| Huang  2010 | >65 | No | Mean age 71.5 (SD=0.64); 48% female (based on completers) | 261 | Group Tai Chi (3 times a week for 5 months) + fall education | Usual care |
| Faes  2011 | Prior fall | Yes | Mean age 78.3 (SD 7.0); 70% female | 33 | Mobility and functional training (2 times weekly for 5 weeks) + psychological training | Usual care |
| Huang  2011 | ≥60; community dwelling | No | 59% female | 186 | Group tai Chi (5 times a week for 8 weeks) + 8 group sessions cognitive training | Usual care |
| Spink  2011 | >65; foot pain; increased risk of falling | Yes | Mean age 73.9 (SD 5.9); 69% female | 305 | Footwear advice/provision + exercises (3 times a week for 6 months) + foot orthoses | Usual care |
| Frieberger  2012 | ≥70; previous falls or fear of falling; community dwelling | Yes | Mean age 76.2 (SD 4.2); 45% female | 153 | Group exercise (twice weekly for 4 months) + home exercises + fall education + cognitive training | No treatment |
| Neelemaat 2012 | ≥ 60; inpatient; malnourished | Yes | Mean age 74.5 (SD 9.5); 55% female | 210 | Vitamin D3 400IU + 500mg calcium daily + nutritional supplements whilst inpatient and on return home | Usual care whilst and inpatient. No nutritional support following discharge home |
| Sambrook 2012 | >70; care home resident | Yes | Mean age 86.4 (SD 6.7); 71% female | 410 | 600mg calcium carbonate + sunlight exposure (30 to 40 minutes, 5 days a week for a year) | Leaflet on Vitamin D deficiency and usual care for their institution |
