# Supplementary material for: Multiple component interventions for preventing falls and fall-related injuries among older people: systematic review and meta-analysis
Source: BMC Geriatr. 2014 Feb 5;14:15. doi: 10.1186/1471-2318-14-15 (PMC3928080; doi:10.1186/1471-2318-14-15)
Supplement: Additional file 2 — Risk of Bias of Included Studies. [file 1471-2318-14-15-S2.docx]

Risk of Bias of Included Studies

|  | Random sequence generation – selection bias | Allocation concealment – selection bias | Blinding of participants and personnel (performance bias) | Blinding of outcome assessment (falls)- detection bias | Blinding of outcomes (fractures) – detection bias | Incomplete outcome data – attrition bias | Selective reporting – reporting bias | Other (assessment of falls – recall bias) |
| --- | --- | --- | --- | --- | --- | --- | --- | --- |
| Steinberg 2000 |  |  |  |  | NA |  |  |  |
| Day  2002 |  |  |  |  | NA |  |  |  |
| Schnelle 2003 |  |  |  |  | NA |  |  |  |
| Clemson 2004 |  |  |  |  | NA |  |  |  |
| Campbell 2005 |  |  |  |  |  |  |  |  |
| Shumway-Cook 2007 |  |  |  |  | NA |  |  |  |
| Swanenburg 2007 |  |  |  |  | NA |  |  |  |
| Zijlstra  2009 |  |  |  |  | NA |  |  |  |
| Bischoff-Ferrari 2010 |  |  |  |  |  |  |  |  |
| Holmgren 2010 |  |  |  |  | NA |  |  |  |
| Huang  2010 |  |  |  |  | NA |  |  |  |
| Faes  2011 |  |  |  |  | NA |  |  |  |
| Huang 2011 |  |  |  |  | NA |  |  |  |
| Spink  2011 |  |  |  |  |  |  |  |  |
| Freiberger  2012 | 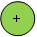 |  |  |  | 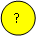 |  |  |  |
| Neelematt  2012 | 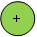 | 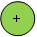 |  | 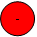 | NA | 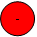 |  |  |
| Sambrook  2012 | 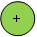 | 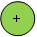 |  | 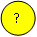 | NA | 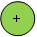 | 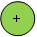 | 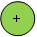 |

Key: + Low risk of bias; - High risk of bias; ? Unclear risk of bias; NA data not collected on fractures as part of study
